# Coat Proteins of the Novel Victoriviruses FaVV1 and FaVV2 Suppress Sexual Reproduction and Virulence in the Pathogen of Fusarium Head Blight

**DOI:** 10.3390/v16091424

**Published:** 2024-09-06

**Authors:** Shulin Cao, Xiaoyue Yang, Lele Xia, Xing Zhang, Haiyan Sun, Yuanyu Deng, Yan Shu, Aixiang Zhang, Huaigu Chen, Wei Li

**Affiliations:** 1 Institute of Plant Protection, Jiangsu Academy of Agricultural Sciences, Nanjing 210014, China; caoshulin@jaas.ac.cn (S.C.); yangxy_1999@163.com (X.Y.); smqxsy@126.com (L.X.); zxchinacn@163.com (X.Z.); sunhaiyan8205@126.com (H.S.); dengyy100@jaas.ac.cn (Y.D.); syan0306@foxmail.com (Y.S.); zhangaixiang2003@163.com (A.Z.); huaigu@jaas.ac.cn (H.C.); 2 Department of Plant Pathology, College of Plant Protection, Nanjing Agricultural University, Nanjing 210001, China; 3 Jiangsu Co-Innovation Centre for Modern Production Technology of Grain Crops, Yangzhou University, Yangzhou 225009, China

**Keywords:** fusarium head blight, victorivirus, mycovirus, coat protein, sexual reproduction, pathogenicity

## Abstract

Fusarium head blight (FHB), a disease inflicted by *Fusarium graminearum* and *F. asiaticum*, poses a growing threat to wheat in China, particularly in the face of climate change and evolving agricultural practices. This study unveiled the discovery of the victorivirus FgVV2 from the *F. asiaticum* strain F16176 and comprehensively characterized the function of the two victoriviruses FaVV1 and FaVV2 in virulence. Through comparative analysis with a virus-free strain, we established that these mycoviruses markedly repress the sexual reproduction and pathogenicity of their fungal hosts. Furthermore, we synthesized the coat protein (CP) genes *CP1* from FaVV1 and *CP2* from FaVV2, which were fused with the green fluorescent protein (GFP) gene and successfully expressed in *Fusarium* strains in wild-type isolates of *F. asiaticum* and *F. graminearum*. Similar to virus-infected strains, the transformed strains expressing CPs showed a significant decrease in perithecia formation and pathogenicity. Notably, CP2 exhibited a stronger inhibitory effect than CP1, yet the suppression of sexual reproduction in *F. graminearum* was less pronounced than that in *F. asiaticum*. Additionally, the pathogenicity of the *F. asiaticum* and *F. graminearum* strains expressing CP1 or CP2 was substantially diminished against wheat heads. The GFP-tagged CP1 and CP2 revealed distinct cellular localization patterns, suggesting various mechanisms of interaction with the host. The findings of this study provide a significant research foundation for the study of the interaction mechanisms between FaVV1 and FaVV2 with their hosts, as well as for the exploration and utilization of fungal viral resources.

## 1. Background

Fusarium head blight (FHB), caused by *Fusarium graminearum* and other *Fusarium* species in wheat, barley, and some other cereal crops, is an economically significant disease worldwide [1]. The pathogens not only cause severe yield losses but also produce mycotoxins such as deoxynivalenol (DON) and nivalenol (NIV), which are considered threats to human and animal health [2]. In China, *F. graminearum* and *F. asiaticum* are the predominant pathogens causing FHB, with an increase in wheat epidemics since 2010 [3]. Currently, chemical fungicides are the primary means of preventing and controlling FHB, but effective biological control strategies are lacking [4].

Mycoviruses are widespread in all kinds of filamentous fungi and oomycetes. Some viruses can reduce the pathogenicity of their host fungi and are considered new resources for the prevention of plant fungal diseases [5,6,7,8]. To date, many viruses have been reported from *F. graminearum*, the pathogen of FHB. Among them, Fusarium graminearum virus 1 (FgV1), Fusarium graminearum virus-ch9 (FgV-ch9), Fusarium graminearum hypovirus 2 (FgHV2), and Fusarium graminearum gemytripvirus 1 (FgGMTV1) cause hypovirulence of their host [9,10,11,12,13]. Recent studies have reported that some of the proteins encoded by mycoviruses play important roles in interactions between viruses and fungi. The structural protein (P3) of FgV-ch9 affects the transcript level of a virus response gene (*vr1*) and causes virus infection-like symptoms in *F. graminearum* [14]. The ORF2 protein (pORF2) of FgV1 can suppress the transcription of the *FgDICER* and *FgAGO* genes and limit the antiviral RNA-silencing response of host fungi [15].

*F. asiaticum* is the predominant pathogen of FHB in the Yangtze River region, an important wheat-growing area in China [16,17]. Recently, many new mycoviruses have been reported in *F. asiaticum* [18,19]. In 2019, we identified a new victorivirus, Fusarium asiaticum victorivirus 1 (FaVV1), from *F. asiaticum* strain F16176 [18]. The members of the genus *Victorivirus* in the family *Totiviridae* are single, linear dsRNA mycoviruses with a genome length of 4.6–7.0 kb that contain two open reading frames (ORFs) encoding putative coat proteins (CPs)- and RNA-dependent RNA polymerase (RdRp) [5,6,20]. Previous studies reported that the victoriviruses Helminthosporium victoriae virus 190S (HvV190S) and Rosellinia necatrix victorivirus 1 (RnVV1) could suppress the pathogenicity of their host fungi [20,21]. However, the impact of FaVV1 on the biological characteristics of the host *F. asiaticum* is not clear.

In this study, we report another new victorivirus named Fusarium asiaticum victorivirus 2 (FaVV2) from the same strain, F16176. We found that FaVV1 and FaVV2 could be cotransmitted to other *Fusarium* strains and not only suppressed pathogenicity but also affected the sexual reproduction of the new host. Further study indicated that the CPs encoded by FaVV1 and FaVV2 are the key factors that cause virus infection-like symptoms in *F. asiaticum* and *F. graminearum*. Advances in understanding the interaction between mycoviruses and fungi may aid biological pest-control approaches using mycoviruses or viral proteins to prevent future *Fusarium* disease.

## 2. Materials and Methods

### 2.1. Fungal Strains and Culture Conditions

*F. asiaticum* wild-type strains F16176, F0609, and F0980; *F. graminearum* wild-type strain PH-1; and hygromycin B-resistant mutants were stored in sand tubes at −80 °C. All strains and mutants were reactivated at 25 °C on potato dextrose agar (PDA) supplemented with or without hygromycin B (100 µg/mL) for further experiments. All strains used in this study are listed in Table 1.

Sexual reproduction was monitored on carrot agar (CA) plates as described previously, with slight modifications [22]. Reactivated strains were inoculated on CA plates and incubated at 25 °C until the aerial mycelia covered the Petri dishes (approximately 5 to 7 days). Then, 300 µL of autoclaved 2.5% Tween 60 solution was added to the plates, and the mycelia were pressed down using a sterile spoon. The cultures were placed under black lights at 18 °C to encourage perithecia development. Perithecia were observed on the CA surface approximately 2 weeks after incubation. For strains that formed perithecia but failed to produce ascospores 3–4 weeks after fertilization, at least 10 perithecia were examined. Each experiment was repeated at least three times.

### 2.2. Genome Sequence Assembly and Phylogenetic Analysis

The genome sequence of FaVV2 was assembled using the method described previously for FaVV1 [18]. ORFs were predicted using Geneious Prime 2021 [23]. Multiple alignments of the amino acid sequences of the conserved domains were performed using the MAFFT program (https://mafft.cbrc.jp/alignment/server/index.html, accessed on 30 July 2019) [24]. Maximum-likelihood phylogenies were inferred using IQ-TREE [25]. ModelFinder [26] was used to select the best-fit model by using the Bayesian information criterion. IQ-TREE and ModelFinder were performed in the PhyloSuite v1.2.1 program [27]. The display, annotation, and management of phylogenetic trees were performed using the online tool Interactive Tree of Life (https://itol.embl.de/, accessed on 30 July 2019).

### 2.3. Virus Cure and Transmission

The virus-cured strain F16176-5a was obtained via hyphal tip isolation from the host strain. Fungal cultures were grown on 1.5% water agar plates at 25 °C for 4 to 5 days, and colony margins containing only one hypha were cut using a scalpel under a microscope and transferred onto new PDA plates. The virus was transmitted into virus-free strains that contained the hygromycin resistance gene (*hph*) by using the protoplast fusion method [28]. The protoplast suspension volumes of the donor and receptor strains were 5:1. Protoplast fusants were regenerated in 1 mL of TB3 (0.3% yeast extract, 0.3% Bacto peptone, 20% sucrose) overnight and then selected on TB3 agar supplemented with 100 µg/mL hygromycin B. Antibiotic-resistant colonies were screened again on plates supplemented with hygromycin B. The absence of viruses was determined using dsRNA extraction and reverse-transcription polymerase chain reaction (RT–PCR) with specific primer pairs (Table S1), and the F16176 strain was used as a positive control.

### 2.4. Vector Construction and Transformation of F. asiaticum and F. graminearum

The complete cDNA sequences of CP1 (from FaVV1) and CP2 (from FaVV2) were synthesized and cloned and inserted into pUC57 vectors using GenScript (GenScript Biotech, Nanjing, China) based on the genomes of FaVV1 and FaVV2. From the vectors pUC57-CP1 and pUC57-CP2, the *CP1* and *CP2* genes were amplified using specific primer pairs with recombinant adaptors and inserted into *Smi*I and *Xho*I-digested pDL2 [29], respectively, using the ClonExpress_II One Step Cloning Kit (Vazyme Biotech, Nanjing, China). CP1/CP2 fusion constructs (CP1-GFP, CP2-GFP) with GFP were generated. The pDL2 vector length was 7613 bp, carrying the Rp27 promoter, GFP marker, and hygromycin resistance gene; it also contained the *Saccharomyces cerevisiae TRP1* gene, the *S. cerevisiae* 2 μm origin of replication, and the *Neurospora crassa* β-tubulin transcription terminator region, and was kindly provided by Dr. Huiquan Liu from Northwest A&F University and Jin-rong Xu from Purdue University [28,30]. *CP* genes were fused with the GFP gene regulated by a strong constitutive RP27 promoter.

The constructed plasmids pDL2-CP1 and pDL2-CP2 were transformed into protoplasts of the wild-type *Fusarium* strains, as previously described [31]. Transformants expressing CP1- or CP2-GFP were identified by PCR and examined for GFP signals via Nikon E400 epifluorescence microscopy.

### 2.5. Virulence Assays

The virulence of strains on wheat heads of the cultivar Yangmai158 was assayed using a previously described method [32]. For each strain, 10 wheat heads were inoculated for analysis. The number of spikelets with symptoms of disease was counted at 14, 21, and 28 days post inoculation. Virulence was estimated according to the mean number of diseased spikelets.

## 3. Results

### 3.1. Another New Victorivirus in the F. asiaticum Strain F16176

**Figure 1:**
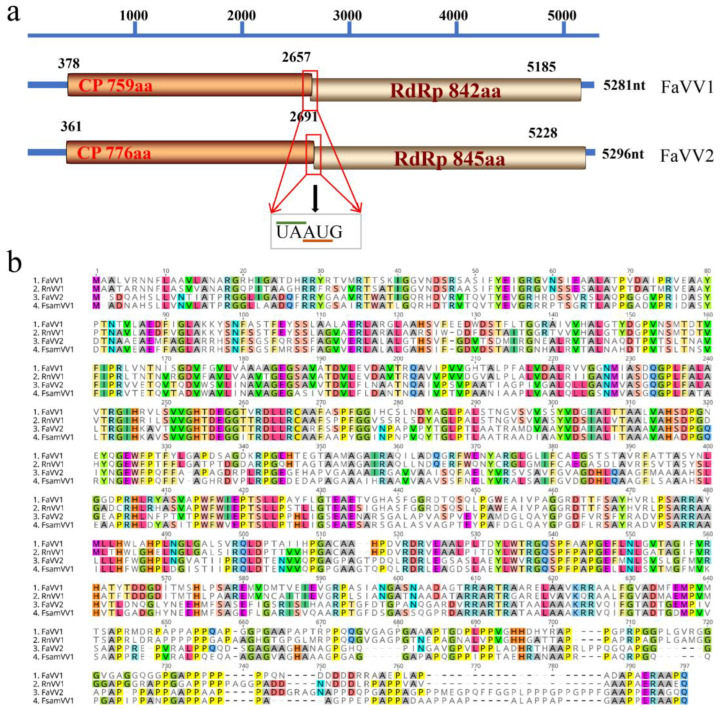
(**a**) Genome organization of FaVV1 and FaVV2. The long rectangular box represents the ORFs of CP and RdRp. The amino acid numbers of the proteins are indicated on the box, and the nucleotide positions of the initiation and termination codons are indicated above the box. The location of the overlapping pentanucleotide “UAAUG” is shown in the figure. (**b**) Multiple-sequence alignment of FaVV1 and FaVV2 CP motifs with those of selected viruses in the genus *Victorivirus*. RnVV1, Rosellinia necatrix victorivirus 1; FsamVV1, Fusarium sambucinum victorivirus 1.

In addition to FaVV1 [18], another new victorivirus was found from *F. asiaticum* strain F16176, which was named Fusarium asiaticum victorivirus 2 (FaVV2). The genome of FaVV2 (GenBank accession no.: MZ042926) is 5296 nucleotides long with a G + C content of 65.2% and consists of a 360 nt 5′-UTR, 2331 nt CP ORF, 2538 nt RdRp ORF, and 67 nt 3′-UTR. The pentanucleotide “UAAUG” overlapped between the CP and RdRp ORFs. These two ORFs putatively encode polypeptides of 776 and 845 aa with calculated molecular masses of 80.24 kDa and 92.00 kDa, respectively (Figure 1a). The genome structure of FaVV2 is similar to that of FaVV1; however, the identities of the CP and RdRp domains between these two viruses are 41.88% and 41.82%, respectively. The CP and RdRp domains of FaVV2 are most similar (74.90% and 72.90% identical, respectively) to those of Fusarium sambucinum victorivirus 1 (FsamVV1) [33] (Mizutani et al., 2021) (Figure 1b).

**Figure 2:**
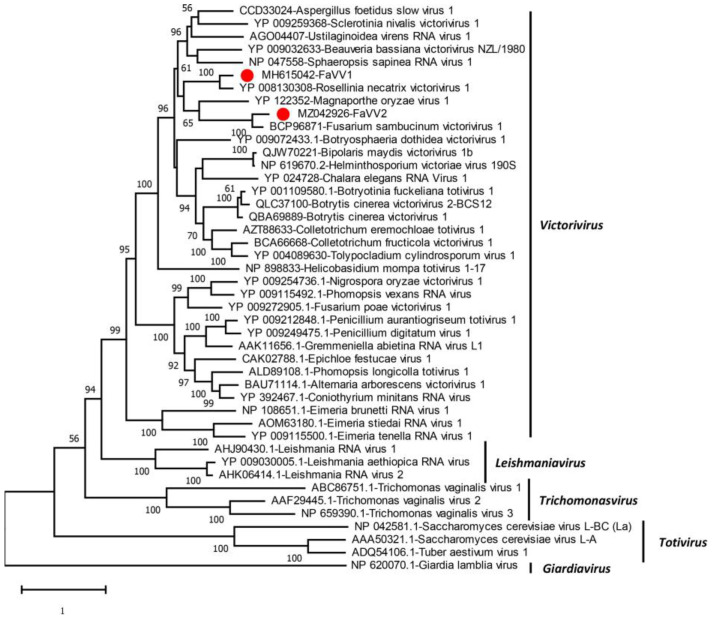
ML phylogenetic tree constructed based on the amino acid sequences of putative RdRp regions of viruses in the family *Totiviridae* with the best-fit model LG + G + I + F. The numbers at the nodes represent bootstrap values above 50%. The RdRp sequence of Giardia lamblia virus (NP_620070) was used as an outgroup. The GenBank accession numbers are placed before the names of the viruses.

Phylogenetic analysis based on the RdRp aa sequences of the viruses in the family *Totiviridae* was performed (Figure 2). According to the ML tree, FaVV2 clustered with FsamVV1 with 100% bootstrap support. The FaVV1, FaVV2, RnVV1, FsamVV1, MoV1, and HmTV1 viruses from fungi were clustered in a clade with relatively low support (52%). Based on the CP and RdRp aa sequence identities and phylogenetic analysis, FaVV2 represents a new victorivirus belonging to the Fusarium sambucinum victorivirus 1 species in the *Victorivirus* genus.

### 3.2. FaVV1 and FaVV2 Suppress the Sexual Reproduction and Virulence of Their Host Fusarium Strains

**Figure 3:**
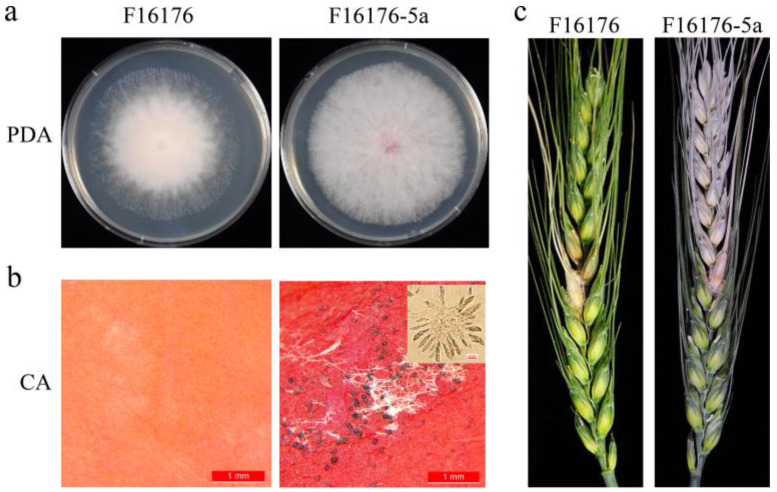
(**a**) Colony morphology of the F16176 and F16176-5a strains on PDA plates at 25 °C for 5 days. (**b**) Sexual reproduction on CA plates (fertile perithecia and ascospores are displayed). (**c**) Pathogenicity on wheat heads.

On CA plates, strain F16176 could not produce perithecium; however, the virus-cured strain F16176-5a produced fertile perithecia and ascospores (Figure 3). At 14 dpi in the infection assay, the average numbers of infected spikelets of F16176 and F16176-5a were 1.6 and 5.9, respectively. The growth rates of these two strains were similar; nevertheless, the aerial hyphae of F16176-5a were thicker than those of F16176 (Figure 3).

**Figure 4:**
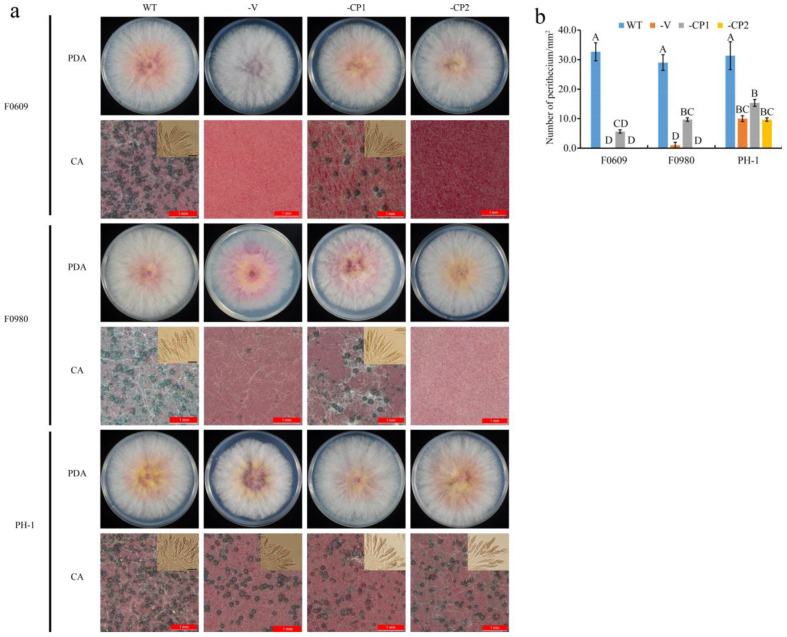
(**a**) Observation of colony growth on PDA plates and production of perithecia and ascospores on carrot medium (ascospores). Scale bar = 20 μm. (**b**) Statistical analysis of perithecia numbers in different strains. Different letters in bars of the same color indicate significant differences at the *p* < 0.01 level according to the Least Significant Difference (LSD) test. WT, wild-type strains; -V, virus (FgVV1, FgVV2)-infected strains; -CP1 and CP2, transformants expressing the coat protein 1 and coat protein 2 strains. The bar chart displays the number of perithecia produced per mm^2^ on CA plates by strain (*p* < 0.01).

**Figure 5:**
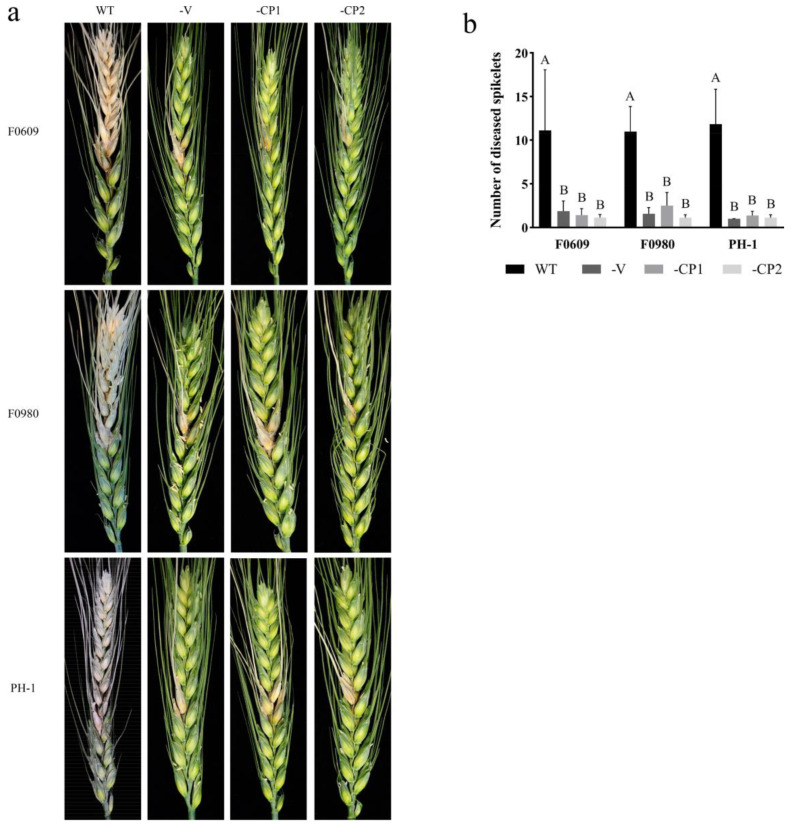
Analysis of wheat heads infection. (**a**) Pathogenicity assessment of *F. asiaticum* and *F. graminearum* on wheat heads. (**b**) Statistics of the diseased spikelets. WT, wild-type strains; -V, virus-infected strains; -CP1 and -CP2, transformants expressing the coat protein 1 and coat protein 2 strains. The bar chart shows the average number of diseased spikelets at 28 days post inoculation. Different letters in bars of the same color indicate significant differences at the *p* < 0.01 level according to the Least Significant Difference (LSD) test.

Using the protoplast fusion method, the FaVV1 and FaVV2 viruses were transmitted to the *F. asiaticum* strains F0609 and F0980 and the *F. graminearum* strain PH-1. Based on the RT–PCR (Figure S1) and dsRNA extraction (Figure S2) results, both the FaVV1 and FaVV2 viruses were successfully cotransferred to the new fungal hosts. However, whether we performed virus removal through protoplast regeneration and hyphal tip isolation methods or virus transmission through protoplast fusion and confrontation culture methods, no instances of the transmission of a single virus, either FaVV1 or FaVV2, without its counterpart were observed. We observe no less than 50 transformants for each method. On CA plates, the sexual reproduction capabilities of these newly infected strains were examined. The *F. asiaticum* F0609-V strain was incapable of perithecium formation, and the *F. asiaticum* F0980-V strain produced a minimal number of small, infertile perithecia. Therefore, we conclude that the *F. asiaticum* strains F0609-V and F0980-V infected with the FaVV1 and FaVV2 viruses are unable to produce ascospores. In contrast, the *F. graminearum* strain PH-1-V was able to generate normal-sized fertile perithecia, albeit at about 50% reduced density compared to the wild-type strain (Figure 4). Despite the differences in sexual reproduction, the growth rates and colony characteristics of the newly infected strains remained consistent with those of the wild-type strains (Figure 4). Compared to their wild-type counterparts, all virus-infected strains demonstrated a significant reduction in pathogenicity (Figure 5). These results demonstrate that the virus may exhibit different effects on different hosts at various stage.

### 3.3. Heterologous Expression of Viral CP Proteins Elicits Virus Infection-like Symptoms

The G + C contents of the *CP1* and *CP2* genes were relatively high, with percentages of 68.1% and 69.7%, respectively, and both of these genes contained poly C sequences at the 5′ end. The attempt to amplify the full-length CP genes utilizing the reverse-transcription products derived from the viral genomic sequences was unsuccessful. Consequently, we artificially synthesized these two genes to facilitate the creation of the transformation vector.

The CP1- and CP2-GFP (green fluorescent protein) fusion constructs were generated and transformed into the Fa strains F0609 and F0980, as well as the Fg strain PH-1, through PEG-mediated protoplast transformation. Both CP1 and CP2 were successfully expressed in the respective transformants (Figure S3); three to five transformants with the same phenotype were obtained for each strain, and one of them is described below for each strain. As a result, the *F. asiaticum* strains F0609-CP1 and F0980-CP1 were capable of producing fertile perithecia, albeit at a reduced density compared to the wild-type strains when cultivated on CA plates. In contrast, the F0609-CP2 and F0980-CP2 transformants were completely incapable of perithecium formation (Figure 4). For the *F. graminearum* transformants, both PH-1-CP1 and PH-1-CP2 were found to produce a reduced number of fertile perithecia compared to the wild-type strains. However, the PH-1-CP2 transformant exhibited an even lower perithecia count than its PH-1-CP1 counterpart (Figure 4). Consequently, the expression of CP2 affects the sexual reproduction of the host in both *F. asiaticum* and *F. graminearum* strains, which is analogous to its effect on the virus-infected strains, whereas the influence of CP1 on sexual reproduction in the *F. asiaticum* strain may be less potent than that in a virus-infected strain. As shown in Figure 3, the growth rates and colony morphology of the transformants remained largely indistinguishable from those of the wild-type strains (Figure 4). Nevertheless, a significant reduction in virulence was observed in all virus-infected and CP-expressing transformants compared to their wild-type counterparts (Figure 5). These results indicate that the virus-encoded CPs play a pivotal role in the virus’s virulence towards the host, thereby directly influencing both the sexual reproduction and the pathogenicity of the host fungi. Specifically, CP2 demonstrated a more potent effect on the sexual reproduction of the native host compared to CP1.

**Figure 6:**
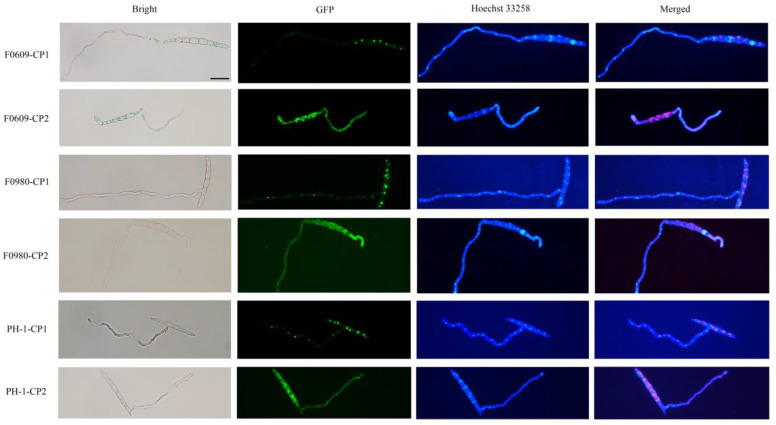
Visualization of CP1 and CP2 protein expression in *F. asiaticum* and *F. graminearum*. Bright, standard light microscopy; Hoechst 33258, fluorescence staining for nuclei; GFP, monitoring the expression and localization of GFP-tagged CP1 and CP2 proteins; Merged, overlay of fluorescence images (red indicates the location of GFP).

Subcellular localization examination revealed that CP1 exhibited a punctate distribution of GFP fluorescence in both the conidia and hyphae, suggesting a concentration within particular cellular structures or organelles. This pattern was consistent across CP1 transformants, suggesting a potential aggregation or specific targeting of CP1. Unlike with CP1, GFP fluorescence in CP2 transformants displayed a diffuse distribution in the cytoplasm, indicating a uniform presence throughout the cell, which seems to be an alternative localization strategy that complements the localization of CP1. Hoechst 33258 staining results show that, neither CP1 nor CP2 was present in the nucleus. This difference in fluorescence patterns between CP1 and CP2 transformants underscores the unique cellular roles and interactions of these viral coat proteins (Figure 6).

## 4. Discussion

With climate change progressing and the production of straw being promoted, the occurrence area and frequency of moderate to severe epidemics of wheat FHB in China have increased recently [3]. The threat of FHB to wheat production in China has become more serious, and alternative prevention and control strategies are urgently needed. The primary infection sources of FHB are ascospores produced by the sexual reproduction of *Fusarium* species [3]. This species can thus be used as a control measure by inhibiting the sexual reproduction of pathogens to cut off the source of primary infection.

In this study, a new victorivirus named FaVV2 was obtained from *F. asiaticum* strain F16176. Phylogenetic analysis based on the RdRp aa sequences of the viruses in the family *Totiviridae* indicated that FaVV2 is a new member of the *Victorivirus* genus belonging to the FsamVV1 family (Figure 1 and Figure 2). To date, two species of victorivirus, FaVV1 and FaVV2, with similar genome sizes have been found from this fungi. Although FaVV1 and FaVV2 are from the same *F. asiaticum* strain, the pairwise identity match of the amino acid sequences of the CP and RdRp domains between these two viruses was less than 50%. According to the phylogenetic tree, FaVV1 clustered with RnVV1, and FaVV2 clustered with FsamVV1 first and then with MoV1. These five fungal viruses clustered in a single clade, but the bootstrap value was less than 50% (Figure 1 and Figure 2). All of the evidence indicates that there is a strong genetic relationship between FaVV1 and FaVV2 and that they may have different evolutionary pathways.

Although FaVV1 and FaVV2 coexist within the same host, there appears to be little genetic exchange between them. However, during horizontal transfer between strains, both viruses are consistently transferred together, suggesting some form of association. This phenomenon may indicate that despite their limited genetic interaction, FaVV1 and FaVV2 probably rely on or facilitate each other’s transmission between hosts. The nature of this relationship remains unclear and may involve specific mechanisms within host cells or environmental conditions that promote the concurrent spread of both viruses.

The majority of mycoviruses exert no significant influence on the biological characteristics of their fungal hosts. However, those mycoviruses capable of attenuating the pathogenicity of these fungi hold considerable promise for biological control strategies [5,6,7,8]. Numerous viruses have been identified within *F. asiaticum* and *F. graminearum*, the causative agents of FHB in China. Among these, several have demonstrated the capacity to mitigate the pathogenicity of their fungal hosts, as reported in recent studies [10]. However, only a few viruses can affect the sexual reproduction of *Fusarium* strains. In this study, the *F. asiaticum* strain F16176 infected with FaVV1 and FaVV2 lost its ability to reproduce sexually, but the virus-cured strain F16176-5a restored the development of perithecia (Figure 3). These results indicate that these viruses may be responsible for suppressing the sexual reproduction of F16176. Therefore, we further transferred the FaVV1 and FaVV2 viruses to other *F. asiaticum* and *F. graminearum* strains. The *F. asiaticum* strains F0609-V and F0980-V could not produce fertile perithecia, and the *F. graminearum* strain PH-1-V produced fertile perithecia, but the density of the perithecia had clearly decreased (Figure 4). These results further confirm that the victorivirus FaVV1 and FaVV2 could suppress the sexual reproduction of *F. asiaticum* and *F. graminearum*. However, the different degrees of inhibition between *F. asiaticum* and *F. graminearum* indicate that the ability of FaVV1 and FaVV2 to inhibit the sexual reproduction of the host may be species-specific.

The ascospores produced by the sexual reproduction of *F. asiaticum* and *F. graminearum* are the primary infection source of wheat head blight, and thus inhibiting the sexual reproduction of the pathogen can reduce the number of primary infection sources. This is highly important for the prevention and control of wheat head blight [3]. In this study, the newly identified FaVV1 and FaVV2 viruses were observed to significantly reduce the pathogenicity of their fungal hosts (Figure 5). Additionally, these viruses suppressed the sexual reproduction of the host, leading to a reduction in the quantity of ascospores, which serve as primary sources of infection (Figure 4). This discovery underscores the promising biocontrol capabilities of FaVV1 and FaVV2 against FHB.

The capsid protein of numerous plant viruses is recognized for its multifunctionality and plays a pivotal role across various stages of viral infection, including invasion, uncoating, protein synthesis, RNA replication, viral movement, symptom manifestation, transmission, and engagement with host cellular factors [34,35]. To elucidate the mechanisms by which the FaVV1 and FaVV2 viruses modulate the pathogenicity and sexual reproduction of their fungal hosts, we engineered a construct in which the viral CP genes were fused to GFP and introduced this construct into fungi. Consequently, the CP1 and CP2 of FaVV1 and FaVV2, respectively, were efficaciously expressed in both *F. asiaticum* and *F. graminearum*, allowing for the investigation of their influence on host biology.

This study has conclusively demonstrated that the mutants of both *F. asiaticum* and *F. graminearum* engineered to express the CPs of the two viruses not only experienced a decrease in pathogenicity but also had their sexual reproductive capabilities compromised. In terms of reducing pathogenicity, there was no significant difference between CP1 and CP2. However, in regard to influencing sexual reproduction, CPs affected the two fungal species differently, with a more significant impact on their native host, *F. asiaticum*. This highlights the crucial role of virus-encoded CPs as key regulators of sexual reproduction in these fungi. Among the two coat proteins, CP2 had a stronger inhibitory effect than CP1. Specifically, the CP1-expressing *F. asiaticum* mutants exhibited an approximately 70% decrease in perithecia production, while the CP2-expressing mutants showed a complete absence of perithecia formation (Figure 6).

Interestingly, GFP fluorescence localization revealed that the subcellular sites of CP1 and CP2 in fungal cells were different. The CP1 protein appears to be localized to specific organelles, while CP2 exhibits a dispersed distribution throughout the cell (Figure 5). These findings suggest that FaVV1 and FaVV2 occupy different niches within fungal hosts, potentially indicating distinct mechanisms of interaction between viruses and their hosts. This may imply that FaVV1 and FaVV2 followed relatively independent evolutionary trajectories, leading to a distant genetic relationship between them. However, during the transfer between fungal strains, both viruses are invariably transferred together, with no instances of single virus transfer observed, suggesting a connection between the two. This paradoxical observation hints at an underlying interdependence or cooperative mechanism during horizontal transmission, despite their apparent genetic divergence.

Despite the fact that the CPs of FaVV1 and FaVV2 interact with their fungal hosts, and affect the pathogenicity and sexual reproduction of the fungi, the mechanisms of these CP–host interactions remain unclear. In further research, we look forward to clarifying the interaction mechanisms between FaVV1 and FaVV2, identifying their molecular targets, meticulously studying how these proteins integrate into the host’s cells, and determining the subsequent effects on the host’s reproductive and pathogenic traits. These studies will characterize the mechanisms of the fungal viruses FaVV1 and FaVV2 in the host fungi, and lay the foundation for the use of fungal viruses to control the primary infection and disease occurrence of wheat scab.

## Figures and Tables

**Table 1:** List of *F. asiaticum* and *F. graminearum* strains used in this study.

Strain	Viruses or Viral CP Content	Background Strain	Antibiotic Resistance
F16176	FaVV1, FaVV2	F16176	
F16176-5a		F16176	
F0609		F0609	
F0609R		F0609	hyg
F0980		F0980	
F0980R		F0980	hyg
PH-1		PH-1	
PH-1R		PH-1	hyg
F0609-V	FaVV1, FaVV2	F0609R	hyg
F0980-V	FaVV1, FaVV2	F0980R	hyg
PH-1-V	FaVV1, FaVV2	PH-1R	hyg
F0609-CP1	CP1	F0609	hyg
F0609-CP2	CP2	F0609	hyg
F0980-CP1	CP1	F0980	hyg
F0980-CP2	CP2	F0980	hyg
PH-1-CP1	CP1	PH-1	hyg
PH-1-CP2	CP2	PH-1	hyg

## Data Availability

Data are contained within the article.

## Supplementary Materials

The following supporting information can be downloaded at https://www.mdpi.com/article/10.3390/v16091424/s1, Figure S1: RT-PCR detection of viral *CP* and *RdRp* genes in receptor strains of *F. asiaticum* (F0609 and F0980) and *F. graminearum* (PH-1); Figure S2: Detection of dsRNA extracted from *F. asiaticum* (F0609 and F0980) and *F. graminearum* (PH-1) after the transfer of FaVV1 and FaVV2. Figure S3: PCR detection of the virus *CP1* and *CP2* genes after transformation into *F. asiaticum* (F0609 and F0980) and *F. graminearum* (PH-1); Table S1: Primers used in this study.
