# Supplementary material for: Coat Proteins of the Novel Victoriviruses FaVV1 and FaVV2 Suppress Sexual Reproduction and Virulence in the Pathogen of Fusarium Head Blight
Source: Viruses. 2024 Sep 6;16(9):1424. doi: 10.3390/v16091424 (PMC11437513; doi:10.3390/v16091424)
Supplement: Supplementary file 1 [file viruses-16-01424-s001.zip › viruses-3145896-supplementary.pdf]

Supplementary file

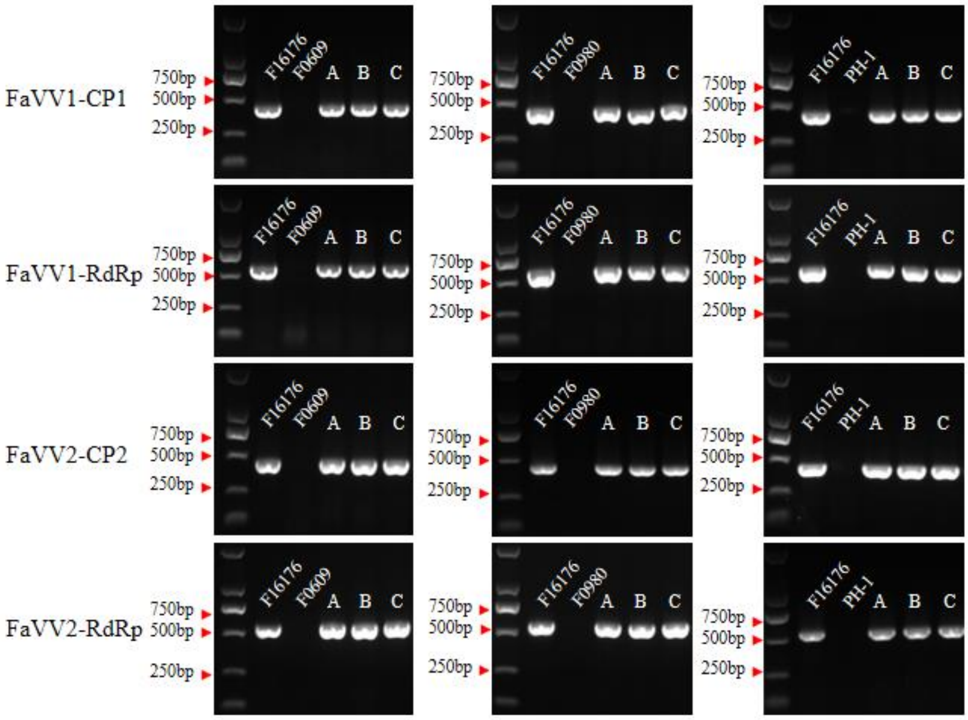

Figure S1. RT-PCR detection of viral *CP* and *RdRp* genes in receptor strains of *F. asiaticum* (F0609 and F0980) and *F. graminearum* (PH-1). Three candidates (A, B, and C) from each strain were selected for virus detection. F16176, positive control; wild-type strains F0609, F0980, and PH-1, negative controls. The primers used for detection were *FaVV1-CP1*, LW123/LW124; *FaVV1-RdRp*, LW127/LW128; *FaVV2-CP2*, LW133/LW134; and *FaVV2-RdRp*, LW129/LW130.

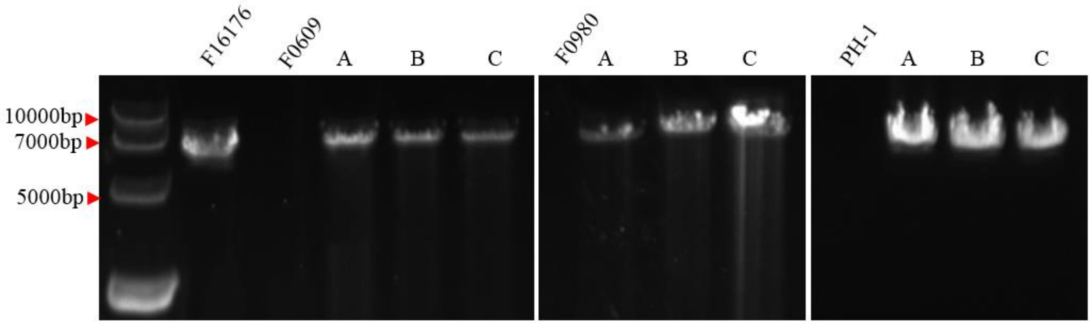

Figure S2. Detection of dsRNA extracted from *F. asiaticum* (F0609 and F0980) and *F. graminearum* (PH-1) after the transfer of FaVV1 and FaVV2. Three candidates (A, B and C) from each strain were selected for detection. F16176, positive control; wild-type strains F0609, F0980, and PH-1, negative controls.

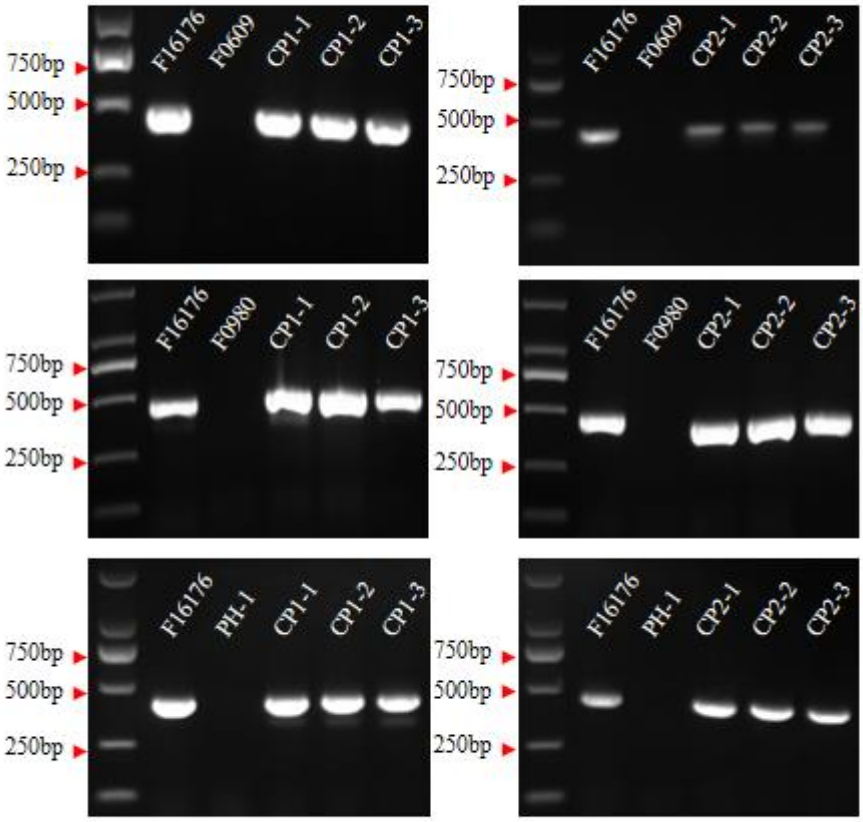

Figure S3. PCR detection of the virus *CP1* and *CP2* genes after transformation into *F. asiaticum* (F0609 and F0980) and *F. graminearum* (PH-1). Three transformants from each strain were selected for detection. F16176, positive control; wild-type strains F0609, F0980, and PH-1, negative controls. The *CP1* and *CP2* genes were amplified using the primers LW123/LW124 and LW133/LW134, respectively.

Table S1. Primers used in this study.

Primer	Sequence(5'-3')	Description	Product size (nt)
LW123	GTGGGATCCACTGTAGCCTG	Screen for the CP gene of the FaVV1 virus.	443
LW124	CGAAGTCGGCTCAATCCAGA		
LW127	CCTCCACACGGGAGATGATG	Screen for the RdRp gene of the FaVV1 virus.	584
LW128	TTGAAGTAGTCACGTCCGCC		
LW129	TCGGATGATCGCATTTCCGT	Screen for the RdRp gene of the FaVV2 virus.	549
LW130	GATCCGTTTACGCACCAAGC		
LW133	CAGGTGACATACGAGGTCGG	Screen for the CP gene of the FaVV2 virus.	447
LW134	AGCGGCGTTCAGGAATAACA		
LW189	CAGATCTTGGCTTTCGTAGGAAC	Amplify the artificially synthesized CP gene	Based on the
LW190	CACCACCCCGGTGAACAGCTCCTCG	from the pDL2 plasmid	gene size
